# Supplementary material for: Multiple spawning run contingents and population consequences in migratory striped bass Morone saxatilis
Source: PLoS One. 2020 Nov 25;15(11):e0242797. doi: 10.1371/journal.pone.0242797 (PMC7688118; doi:10.1371/journal.pone.0242797)
Supplement: Attachment — Submitted filename: PLOS_One_2018AUG.docx [file pone.0242797.s001.docx]

Review of Secor et al.: “Multiple spawning run behavior and population consequences in migratory striped bass Morone saxatilis ”

**Summary**

In their manuscript, Secor et al. describe their identification of two spawning subgroups within the Hudson River Estuary population of ocean-migrating striped bass that spawn annually. They suggest that these two subgroups are spatially segregated during spawning and follow similar migratory paths but exhibit different phenologies (i.e., staggered within the spawning season) as they enter and leave their respective spawning grounds. The mean daily latitudes of fish within these spawning subgroups were tracked with acoustic telemetry. Movement patterns of subgroups into the spawning reaches were then defined (upper and lower spawning runs) and time series differences analyzed using dynamic time warping. Individuals are proposed to maintain membership within established groups over time, based on 2 years of recapture data (2017-2018), where characterization of the upper versus lower spawning run subgroup was based on the 2017 spawning run. The lower spawning run occurred earlier (entered the study area and left the study area earlier) than the upper spawning run in 2017, but not 2018. They also find that these two subgroups follow similar routes along the Atlantic shelf outside of the spawning season. Moreover, the authors argue that different mortality rates between the subgroups help to stabilize the larger population dynamics.

**Major comments**

I think this is an interesting study that characterizes patterns of movement behavior for striped bass within the Hudson River Estuary and on the coastal shelf, and provides some support for spatial segregation of an upper and lower run during spawning. The manuscript was written well, and generally easy to understand. Survival analyses and conclusions from the survival analyses seemed appropriate (assuming that lower run fish were adequately detected, which was not clear to me based on Lines 149-150). The authors did a good job relating the increased mortality rate of the lower spawning group with the earlier (relative to the upper spawning group) entrance into shelf fisheries in 2017. This earlier migration behavior was potentially initiated in response to a steady increase in water temperatures throughout April that also triggered their earlier movement up-estuary to spawn.

A main concern is that the membership of individual tagged fish within the lower spawning run centroid was only consistent across 2017-2018 for 8 of 12 fish. Thus, although it is true that only 11% of individuals were misclassified between the two years overall, all of those misclassified were originally thought to be part of the lower spawning group, with few fish in that group overall. If the study was able to recapture fish in 2019, and there was evidence of continued site fidelity by those lower spawning group individuals, the case for spatially segregated groups with consistent membership would have been much stronger.

In addition, this group seems to have much lower membership overall, so I wonder how much it would be able to contribute to an overall stabilizing portfolio effect. If there is evidence of at times higher spawner abundances in the lower reach than in the upper reach (e.g., based on the decades of monitoring spawning adults mentioned in Lines 118-120), please include in Discussion. It is mentioned in Lines 452-454 that future climate change and warmer springs could lead to favoring the lower run. Please expand on how warmer springs could favor the lower run because it is not clear to me. Addressing these issues would help support the authors proposal that the two spawning runs could contribute to a portolio effect that stabilizes population dynamics for this contingent of striped bass.

**Minor comments**

- Line 116-117: “Spawning adults associated with these reaches are believed to be centered” – what is this belief based on? Observations from the authors?
- Line 117: Should 160-120 be 160-200? However, the lower boundary of the red rectangle in Figure 1 looks like it is greater than 160.
- Line 189: I am not seeing blue and red time series in Figure 2, as described here in the figure caption, just shades of brown.
- Line 213: Can leave out “excluding May”
- Line 293: I see a leveling off in Fig. 6 that starts the beginning of May and extends through mid-May, rather than a plateau only during mid-May
- Line 383-384: The authors included this statement: “Developed as a machine learning tool in speech recognition applications, we believe this is its [dynamic time warping] first application in comparing animal movement patterns.” As I wasn’t familiar with dynamic time warping, I did a quick search to see that it has recently been a suggested approach for analysis of animal movement as demonstrated within Cleasby et al. (2019), and applied to seabirds. I realize this is a recent paper, so understandably missed. I suggest the authors include this reference within the manuscript and adjust their statement accordingly.

References:

Cleasby et al. 2019. Using time-series similarity measures to compare animal movement trajectories in ecology. Behavioral Ecology and Sociobiology **73**.
