# Supplementary material for: Multiple spawning run contingents and population consequences in migratory striped bass Morone saxatilis
Source: PLoS One. 2020 Nov 25;15(11):e0242797. doi: 10.1371/journal.pone.0242797 (PMC7688118; doi:10.1371/journal.pone.0242797)
Supplement: Attachment — Submitted filename: Response to Reviewers_Oct2.docx [file pone.0242797.s002.docx]

We thank the editor and reviewer for their guidance. In the experience of the first author, these are among the most thoughtful and helpful reviews he’s received and have done much to improve the manuscript. Major comment responses are presented by reviewer. Minor responses are collated by where they occur in the manuscript and labeled R1 and R2 for the two reviewers. Our responses are in red, larger revisions are given in italics and their by line location in the clean copy of the manuscript is noted.

**Major Comments**

Reviewer 1

1) Spawning runs vs contingents: Throughout the manuscript (e.g. line 24, 30, 61-63), the authors move back and forth between using the terms spawning run, spawning run contingent, and contingent to describe the different migration phenologies they observed. Given the first author’s original description of contingents in the 1999 paper, I believe that term is most appropriate, but do not believe it should be used synonymously with spawning run. At least within Pacific salmon, spawning runs are commonly used to describe genetically isolated groups, indeed run timing is one dimension used to define ESUs in the US and CUs in Canada, while the authors note that the upper and lower reaches are likely to have substantial genetic exchange. That being said I recognize that the definition of life history terms can vary region to region and taxa to taxa. Thus, my main recommendation would be to clearly define early in the introduction how each term relates to the other. Note that this may impact the manuscript’s title.

Also in the introduction, it would be beneficial to clarify how spawning location, phenology, or both contribute to classifying contingents in this context since temporal or spatial segregation could be used to classify a run, but evidence for each is somewhat ambiguous here (e.g. straying between groups, downstream advection of eggs, similar migration phenologies in one year).

Thanks for these viewpoints, and we agree that terminology is not straightforward, particularly as concepts of multiple spawning runs and the portfolio effect depend in part on a rich salmon literature. As the reviewer states, seasonal spawning runs for Pacific salmon are key units for conservation, which are most rigorously defined through genetics. Still, these are major genetic units, within which multiple movement phenologies occur to various segments within a given river system: groups without strong genetic separation (e.g., Beacham et al. 2014). We consulted a preeminent salmon colleague, who pointed us to clear precedence in terminology, which does support the referee’s views. We think the agnostic term contingents, used to describe behavioral groups works well as a more general term and thus have opted to use the term spawning run contingents or spawning reach contingents. According to the reviewers suggestion we have also opted to use movement phenologies when referencing individual spawning movements. Runs are reserved to describe annual spring runs (thus 2017 and 2018 runs). We have repaired text where these definitions are not consistently applied, including the title. Clarification on terms is provided in the last paragraph of the Introduction:

L85-97.

*To be of ecological consequence, each spawning run contingent should exhibit characteristic migration behaviors, repeat these behaviors across years, and encounter varying mortality regimes. Run contingents are defined as a cycle of directed up-estuary and down-estuary migration behaviors occurring within the Hudson River Estuary, and are classified by both phenology (timing) and spawning reach (up-estuary extent). We leave alone the question of evolutionary and conservation consequences associated with genetic differentiation between contingents [28], and rather examine ecological consequences of their behaviors following their departure. [29, 30]. Through biotelemetry and time series classification using dynamic time warping, we asked; (1) Are characteristic spatiotemporal migration behaviors repeated within successive spring spawning runs of the tagged individuals? (2) Do the same individuals undertake the same contingent behavior between years? (3) Do spawning run contingents undertake the same migration behaviors during non-spawning periods? and, (4) Do mortality rates vary between spawning run contingents?*

2) Dynamic time warping – As the authors note, DTW is not commonly encountered in ecological analyses. Indeed, I had to spend a decent amount of time reviewing the relevant literature to understand what was happening under the hood. The authors briefly introduce the technique in the methods and provide some additional information in the discussion, but I believe an additional short paragraph summarizing the primary references would be beneficial for readers who are curious about the technique.

Thanks to the reviewer for the time spent to dig into DTW. We have re-written the methods paragraph introducing DTW to explain, in an accessible manner, what the algorithm is doing and the implications of such. As well as a description in our own words, we add the citation of Sardá-Espinosa 2019, a vignette for the *dtwclust* R package used in our analysis. We believe this gives a concise overview of the origin of DTW, different algorithms to obtain it, and the procedure used “under the hood”. Following suggestions of the other reviewer, we also note two recent instances of DTW-based clustering in the sea bird migration literature: Cleasby et al. 2019 and Frankish et al. 2020.

The paragraph summarizing DTW has been changed to the following:

L. 192-208

*Individual spawning phenologies were categorized by clustering around median centroids (k-medoids [47]) utilizing dynamic time warping as a time series dissimilarity measure. Dynamic time warping, a machine learning algorithm most often applied in speech recognition classification, was chosen due to its suitability in matching phenomena that are offset in time and magnitude [48, 49]. Starting from the beginning of the time series, dynamic time warping iteratively finds which points are matched with the least cost [50]. This allows matching of multiple-to-one or one-to-multiple points (Fig 2), and reports the cost of “warping” the two time series to be alike in this manner. Compared to Euclidean matching, which necessitates one-to-one matching in time and would calculate dissimilarity between spawning run contingents based on the daily river kilometer distance between each fish, dynamic time warping measures how much an entire spawning phenology trajectory must be stretched or compressed in time to match that of another fish. As such, spawning run phenologies that are alike in shape, extent, and destination, but offset in time are clustered together[51, 52]; Fig 2). The iterative clustering procedure, utilizing dynamic time warping and performed with the dtw[53] and dtwclust [54] packages in R, solved for maximum within-cluster similarity and minimum between-cluster similarity. A synthetic median centroid was extracted from each of two clusters to represent lower- and upper-reach spawning.*

On a related note, I initially struggled to interpret Figure 2, but if adapted, I believe it offers an opportunity to introduce and clarify DTW to readers. First, I would recommend showing the Euclidean plot in the top panel since readers are more likely to be familiar with that distance metric. Second, it would be beneficial to add text insets (e.g., a, b, and c at the beginning, middle, and end of the time series), which are referenced and interpreted in the caption since readers are not fully familiar with the study’s data until the end of the methods, but Fig. 2 is referenced partway through. Finally, I would suggest summarizing what the implications of the Euclidean vs. DTW distance measures are in this simple example (either in the caption or when Fig. 2 is referenced in the text). For completeness, it may be helpful to add a second panel, which shows two time series where Euclidean and DTW estimates provide relatively similar conclusions to contrast visually with the example where they clearly are different. As an aside, in my version the second figure does not have any red or blue, just two shades of brown (I’m using a Mac so perhaps this is a PDF conversion issue?).

We thank the reviewer for the excellent suggestions to improve Figure 2, especially those relating to highlighting crucial stages in the spawning run (river entry, spawning ground arrival, spawning reach exit, river exit) that could not otherwise have been matched using Euclidean methods. Figure 2 caption has been adjusted from blue/red to light/dark brown; this was a holdover from a previous iteration of the figure. For ease in review, we duplicate the revised figure below. Note we opted not to present case for similar patterns between both approaches, as we think the revised figure adequately exemplifies the approach and its potential advantage.

***Fig 2.*** ***Visualization of matching two spawning runs via standard Euclidian (top panel) and dynamic time warping (bottom panel) methods.*** *Spawning runs depicted as light brown (left axis units) and dark brown (right axis units). Time series are vertically offset for ease of visualization. Gray lines display daily positions matched using the respective algorithms). The arrival of each fish (subscript 1, 2) in the river (A) or spawning reach (B), and subsequent exits from the spawning ground (C) and river (D) are shown. Note that dynamic time warping allows these spawning run phases, offset in time, to be matched, while the Euclidean method matches only those points that co-occur in time.*

3) Environmental linkages – I understand the authors interest in identifying drivers of interannual variability in run timing and agree that discharge and temperature are two plausible candidates for consideration. However, I was surprised that the authors describe strong linkages between temperature and phenology in the discussion given their results. First, since the observational unit in this part of the analysis is an entire run (sampling population level response to a shared driver), it is difficult to draw robust conclusions about the relationship between drivers and phenology with only two years of data. Second, only the upper run delayed in 2018 and the 50% migration point was remarkably stable between years despite a 3-4 C difference in temperature (depending on the run). There was also considerable variation within a run, which suggests that even if temperature does cue the run, it likely only explains a relatively small component of the total variability. In other words, evidence of temperature cues seems to be weak and ambiguous and I actually was under the impression that they would discuss the stability of run timing despite interannual differences in temperature

Similarly, it seemed odd to me that temperature at the mid-point of the run was used as a proxy for thermal conditions that may cue both river entry and egress. Since the data suggest considerable variation in the rate at which warming occurs, it seems as though temperatures at the beginning and end of the summer should be correlated with entry and egress, respectively. Finally, I didn’t understand why discharge data were presented in Fig. 6, but not incorporated into the analysis since they appear to show substantial variation between years as well. If I am misinterpreting the authors analyses (which is possible) I would recommend they clarify their hypotheses and methods, and perhaps introduce additional language that emphasizes the limitations of their approach given the data available.

The reviewer correctly identifies a struggle we had in using only two years of spawning run data yet wishing to still investigate the role of temperature in migration behaviors. In revisions, we bring in additional literature to support the key role of temperature in striped bass spawning times (this same literature had been presented in Discussion to discuss how 2-4 C difference in spawning time had consequences to larval production). Flow then becomes an ancillary variable to better understand changes in temperature. (Flow is an important spawning cue in non-tidal riverine systems where striped bass spawn – e.g., Congaree-Wateree system in South Carolina and Sacramento River in California, but in tidal systems, flow has typically been viewed as controlling post spawning success of larvae and juveniles). We’ve added a statement to the Introduction and modified statements elsewhere to better reflect this analysis.

L. 97-99

*Within the limits of two years of spawning phenologies, we also evaluated the influence of temperature, which has been identified as a dominant spawning cue for striped bass [30-32].*

In Methods we made this revision:

L.224-226

*Temperature was examined as a possible driver for the phenology of 2017-defined run contingents, under the premise that the 2017 classifications best represented the two run contingents. Flow was also examined for its influence on temperature.*

The reviewer provides a strong case for greater similarity than differences in spawning run phenology with respect to temperature. We have revised Results and Discussion accordingly.

L.321-330 *The upper reach contingent lagged behind the lower reach contingent, measured as 50% incidence, more so in 2018 than in 2017 (Fig 6). Different responses by continents to temperature may be indicated for the upper reach contingent, which shifted its timing earlier during the warmer year (April 22, 2017) in comparison to the cooler year (April 27, 2018). In contrast the lower reach contingent, exhibited the same date of 50% incidence between years (April 18). With the exception of the lower reach contingent in 2017, a few individuals from all in both years entered the river in early April when mid-river water temperatures were 5-6 °C. In 2017, mean temperatures at 50% incidence were 10.1 and 11.8 °C, respectively for the lower and upper reach contingents. In 2018, 50% incidence occurred at substantially cooler mean temperatures of 6.1 (lower reach contingent) and 8.0 °C (upper reach contingent).*

L.434-450

*Striped bass spawning runs occurred during springtime warming, which ranged 4°C to 23°C across individual spawning phenologies. A slower rate of warming in 2018, was associated with a 5 day lag of the upper reach contingent, but the timing of the lower reach contingent was similar between years. This suggests that spawning run phenologies are influenced, but not tightly cued by temperature. In both years, a minority (<10%) entered early as temperatures ranged 4-8 °C. Entry during winter regime temperatures aligns with historical Hudson River fisheries which targeted fish under ice-cover [22]. Spawning migrations under ice cover have been noted for Canadian systems as well [37]. In both years, the number of fish participating in spawning runs increased rapidly between 10 and 12 °C. Then, most fish departed by the beginning of June as temperatures approached 20 °C. The lower reach contingent likely spawned at lower temperatures than the upper reach contingent with the difference larger in 2018. A recent historical analysis of spawning temperatures in the Hudson River Estuary recorded a range of 11.9 to 23.0 °C[33]. In more intensively studied Chesapeake Bay tributaries, spawning is associated with several-day surges in water temperature within this range [31, 63]. In other literature, 12-20°C bracket viable temperatures for embryo and larval growth and development in the Hudson River [32, 64] and elsewhere [31, 65-67] and in general the most favorable temperatures for larval survival occur between 15 and 19 °C [63], a period of maximum presence by spawning runs in both study years.*

4) Differential mortality – I found the survival data very interesting and agree with the authors that it is consistent with differential mortality among the contingents, which is clearly of management interest. That being said, it’s not clear to me how a moderately earlier ocean entry (~seven days) led to sustained higher rates of mortality over ~two months for the lower reach group. If differential mortality was driven solely by built up recreational harvest then why wouldn’t the difference in survival be restricted to May/June, rather than continue through July (based on Fig 7)? Is it possible these are delayed mortalities from catch-release fisheries, particularly if lower estuary fish are exposed to higher in river harvest effort? Otherwise there must be some difference in exposure to fisheries, or natural mortality mechanisms, through the summer to drive a consistently divergent pattern; however, that doesn’t seem to be the case based on the coastal detections that are presented. More generally, I would caution the authors against emphasizing harvest as the driver of differential mortality unless they can present data distinguishing natural mortality from fisheries mortality within the tag groups (e.g. a larger proportion of lower reach tags returned by harvesters) or it is established that natural mortality for these age classes is truly negligible. If the latter, appropriate references should be added.

The mortality analysis brackets detection times so that the dropped step observed in July corresponds to June mortality. Please recognize also that much like entry, departures do not occur all at once, in both years spread across May and June. Fishing mortality is the dominant source of mortality for these large striped bass, justifying its retention as a focus of the manuscript (NEFSC 2019). We do recognize that our description of pent up demand may have been too speculative and have removed that from Discussion. Further, we emphasize differences between 2017, where exit times and mortality rates differed between spawning run contingents and 2018, when mortality rates and exit times were similar. This too aligns with a shelf fishery that selects emigrating fish as they become available. The interpretation that catch and release could also contribute to losses is insightful. Still because, fishing occurs throughout the Hudson River and is in fact centered more in the upper reach, it would seem unlikely that this would present itself as increased mortality on lower reach fish. We have clarified and modified content in Results and Discussion to emphasize the above points.

L.368-377

*The lower reach contingent experienced a high level of loss during the May-June period in 2017 and 2018 (Z=4.9 10^-3^ d^-1^; Z=7.4 10^-3^ d^-1^), suggesting periods of intense vulnerability (Fig 8). In comparison, the upper reach contingent experienced lower mortality during this period in 2017 (Z=1.0 10^-3^ d^-1^), but similar losses in 2018 (Z=4.5 10^-3^ d^-1^). Annual post-run Kaplan-Meier survival estimates differed between spawning run contingents only for 2017 (p= 0.02; 2018: p=0.7). In 2017, annual post-run survival was estimated at 57.1 ± 10.8% and 82.2 ± 5.7%, for the lower and upper reach contingents, respectively. In 2018, annual post-run survival was similar between contingents (lower: 54.5 ± 15.0%, upper: 51.6 ± 9.0%). Note that the median exit date for the lower reach contingent was 9 days earlier than the upper reach contingent in 2017, and that the two contingents exhibited similar exit dates in 2018 (Table 1).*

L.471-483

*We speculate that by departing the Hudson River and entering coastal fisheries 6-9 d earlier in 2016 and 2017, the lower reach contingent experienced greater fishing mortality than the upper reach contingent. Higher vulnerability would persist as this contingent successively entered New England state fisheries as it migrated northward. In 2018, when little difference occurred in median exit dates, mortality was similar between contingents suggesting similar exposure to these fisheries. Directed fishing in the river itself is largely focused in the upper reach[72], such that any latent mortality associated catch and release of larger spawners would likely bias mortality opposite to observed pattern of contingent mortality. As further circumstantial evidence of higher selectivity on early departing striped bass, consider the “other” (undefined) category, which principally comprised individuals that did not survive until the following spring and therefore could not be included in the clustering procedure. In both 2016 and 2017, other category individuals departed to shelf fisheries at substantially earlier dates and may have been exposed to higher effort resulting in their loss (Figure 7).*

Reviewer 2

I think this is an interesting study that characterizes patterns of movement behavior for striped bass within the Hudson River Estuary and on the coastal shelf, and provides some support for spatial segregation of an upper and lower run during spawning. The manuscript was written well, and generally easy to understand. Survival analyses and conclusions from the survival analyses seemed appropriate (assuming that lower run fish were adequately detected, which was not clear to me based on Lines 149-150). The authors did a good job relating the increased mortality rate of the lower spawning group with the earlier (relative to the upper spawning group) entrance into shelf fisheries in 2017. This earlier migration behavior was potentially initiated in response to a steady increase in water temperatures throughout April that also triggered their earlier movement up-estuary to spawn.

We thank the reviewer for these positive statements and concise summary.

A main concern is that the membership of individual tagged fish within the lower spawning run centroid was only consistent across 2017-2018 for 8 of 12 fish. Thus, although it is true that only 11% of individuals were misclassified between the two years overall, all of those misclassified were originally thought to be part of the lower spawning group, with few fish in that group overall. If the study was able to recapture fish in 2019, and there was evidence of continued site fidelity by those lower spawning group individuals, the case for spatially segregated groups with consistent membership would have been much stronger.

Thanks for this comment. We now highlight higher misclassification of the lower run in Results and Discussion. Text already emphasizes this same point related to fidelity to region of tagging.

L.289-295

*The 2017 and 2018 clustering procedures provided similar individual classications with all but 4 of the 38 individuals that returned in both years (two of the 40 fish returning in 2018 had not returned in 2017) correctly classified (11% misclassification; chi-square test: P<0.001; Figs 4,5). All misclassified individuals in 2018 occurred for the lower reach contingent (4 of 12 individuals; Fig. 5C).*

L.398-401

*Given the level of infidelity between runs (11%), particularly for the lower spawning run (25%), and high dispersal and mixing of embryos and larvae [24], spawning run persistence is unlikely related to genetic lineage.*

In addition, this group seems to have much lower membership overall, so I wonder how much it would be able to contribute to an overall stabilizing portfolio effect. If there is evidence of at times higher spawner abundances in the lower reach than in the upper reach (e.g., based on the decades of monitoring spawning adults mentioned in Lines 118-120), please include in Discussion. It is mentioned in Lines 452-454 that future climate change and warmer springs could lead to favoring the lower run. Please expand on how warmer springs could favor the lower run because it is not clear to me. Addressing these issues would help support the authors proposal that the two spawning runs could contribute to a portolio effect that stabilizes population dynamics for this contingent of striped bass.

In past papers, we have shown that even minority behaviors can contribute to persistence (buffering) when the dominant behavior fails (Kerr et al. 2009; Secor et al. 2009); the key here is non-stationarity. We like the suggestion of referencing egg production to show how the lower run may not always constitute the minority behavior and thank the reviewer for this. We have made related revisions.

L.492-498

*Here, contingents encounter varying mortality and reproductive success but jointly, their outcomes buffer the population under nonstationary mortality regimes. In instances where one contingent is substantially smaller in number, such as indicated here for the lower spawning run contingent, the portfolio effect is diminished, although resilience (persistence) is still enhanced [7, 67]. Still, our sampling frame was curtailed to a single year’s tagging effort and egg densities suggest a more equitable spread between spawning runs during the past several decades (Figure 1).*

Thanks to the reviewer for asking for greater specification on why early spawners might do better during warmer springs. This was indeed not explained. Revisions to Discussion:

L.501-504

*We observed that early departure likely caused higher fishing mortality in the lower reach contingent in 2017, but in a scenario of future climate change and warmer springs [33], reproduction and early survival could be favored in the lower reach owing to reducing the current risk of spawning in cold sub-lethal temperatures [31, 63].*

**Minor comments**

Reviewers combined and ordered by line number: R1=Reviewer 1; R2=Reviewer 2

Line 41 (R1): Replace with “enter” singular.

Replaced as suggested.

Line 43 (R1): Suggest replacing “contributing to” to “potentially stabilizing” given the focus on hypothetical portfolio effects.

Replaced as suggested.

Lines 71-73 (R1): The phrase “centers” implies to me a multimodal distribution of spawning density; however, the egg data in Fig. 1 appear approximately normally distributed suggesting a continuum of spawning habitat rather than distinct spawning areas. That being said, the rationale in lines 116-118 seems reasonable, I would suggest adding a brief version to the figure caption to help confused readers.

We have rephrased text from “centers” to “concentrations,” preferring not to expand text in the figure legend.

Line 79-80 (R1): Suggest re-phrasing to “Points show yearly values of egg density and box plots represent interannual variation”.

Rephrased as suggested.

Line 88-89 (R1): I find the phrase “characteristic migration modes” a little vague. Perhaps specify whether the interest is primarily variation in spatial distribution, temporal distribution, or both? Lines 71-73 suggest the interest is primarily spatial, but the first paragraph, and the bulk of the manuscript, include reference to timing as well.

Rephrased to “characteristic spatiotemporal migration behaviors”.

Line 104 (R1): Strictly speaking where do lower estuary individuals fall on the resident/migratory continuum? Also 126 indicates all individuals were migratory ocean contingent. Since fish were tagged in the upper estuary, where individuals are defined as resident, how was this determined?

Fall tagging in this region has indicated upper HR estuary captured fish would be resident, but this is not the case during spring when the ocean contingent dominates (Wingate and Secor 2007; Gahagan et al. 2015). We would prefer to do this through references without describing past otolith chemistry and telemetry data that shows the resident contingent and lower estuary contingents which can be selectively sampled during fall and the oceanic contingent which is selectively sampled during spring. We’ve added a line to more specifically lead readers to related research.

L. 114-115

*In this study, we target the Oceanic contingent through selection of larger individuals during spring [41].*

Line 116-117 (R2): “Spawning adults associated with these reaches are believed to be centered” – what is this belief based on? Observations from the authors?

Yes, we agree this is rather circuitous. We have restructured the text to be more direct in presenting evidence for these centers.

L.125-128

*Decades of monitoring spawning adults confirm the traditional occurrence of spawners in the 80-100 km and 160-200 km reaches[44]. Egg densities are likely displaced downriver from these spawning centers owing to down-estuary advection of eggs. Proximity to the salt front retains eggs spawned at the lower reach [25].*

Line 117 (R2): Should 160-120 be 160-200? However, the lower boundary of the red rectangle in Figure 1 looks like it is greater than 160.

“160-120” corrected to “160-200”. Colored rectangles in Figure 1 have been revised to align with the rounded river kilometers of spawner occurrence provided in lines 117 – 120, which are contained within the larger 160-200 km region. Note that this adjustment is solely cosmetic and has not affected data analysis, results, or inference.

Lines 128-130 (R1): Any concerns of differential effects of seining vs. electrofishing?

As we saw no differential mortality associated with these releases in 2016 (covered in text – just one fish apparently lost immediately following tagging), we only address the reviewers concerns here. We had nominal concerns regarding the effects of seining vs electrofishing. Seined fish are subject to capture stress and are usually not injured although additional stress and mortality can occur while the catch is being processed. Stress-related death from electrofishing usually occurs within minutes or a few hours after a fish is shocked and larger fish are more likely to be injured than are smaller ones because their larger size makes them more susceptible to electroshock and muscle contractions are more severe in larger fish (Reynolds and Kolz 2012 Fisheries Techniques (pp.305 to 361). Third Edition. Chapter: 8. Publisher: American Fisheries Society). The longer captive time associated with seining techniques likely makes the risks associated with seining more equitable to the risks associated with electrofishing. In a study comparing gillnetting with electrofishing, it was found that electrofishing was less stressful on striped bass than gillnetting and only the fish that had been captured through electrofishing made a full recovery in the 48 hr study period (Harrell and Moline 1992. World Aquaculture Society 23:58-63). Additionally, both electrofishing and haul seining have been used as capture gears for NY’s spawning stock and tagging survey since 1989. During that time period, there has been no significant difference between the return rate of bass tagged through the different gears. Electrofishing was also used as the method of capture for the control group of striped bass in catch and release mortality study done in the Hudson (Millard et al. 2003. Fisheries Management and Ecology 10:295-300).

Line 142 (R1): Duration of holding? ” Fish were held in pre- and post-surgery recovery pens.”

Fish selected for tagging were immediately transferred to a flow-through tank or in-river live well, surgery was performed, and fish were immediately released after exhibiting recovery in the live well. Holding time both pre- and post- surgery was adequate to both ensure healthy condition (<5 minutes each) and to minimize holding stress on the fish. We have added two related statements.

L.142-145

*Fish selected for tagging were immediately transferred to a flow-through tank or in-river live well. Holding time both pre- and post- surgery was adequate to both ensure healthy condition (<5 minutes each) and to minimize holding stress on the fish.*

Line 146 (R1): Adding receivers to Fig 1 would be helpful.

Receivers are represented by circles on the map of Figure 1. Line 145 has been corrected with the exact number of receivers to more-clearly align with the number of circles depicted in Figure 1.

Lines 152-161 (R1): I know that acquiring receiver metadata from tracking networks can be difficult, but if possible, a map showing the distribution of the marine receivers (similar to Secor et al. 2020) would be helpful.

Figure 7 has been updated to display the distribution of marine receivers.

***Fig 7. Atlantic shelf water telemetry detections of Hudson River striped bass spawning runs.*** *Top: Detections through time. Upper and Lower runs are defined by clustering of 2017 spawning run trajectories. The “Other” category are those not included in the 2017 classification owing to tag loss, skipped spawning, or straying. Detections are ordered by latitude of telemetry receiver array (Maine – Coastal Virginia/Chesapeake Bay). ME=Maine estuary and shelf waters, MA=Massachusetts and Cape Cod Bays, LIS=Long Island Sound, NYB (NY Bight)=New York and New Jersey shelf waters, Hudson=Hudson River Estuary; DE=Delaware River and shelf waters, MD=Maryland, VA=Virginia, CH=Chesapeake Bay.*

*Bottom: Distribution of each year’s detections with labeled general location of receiver arrays.*

Line 175 (R1): It might be useful to insert summary statistics on the extent to which individual time series had to be imputed (i.e. mean + SD of number of imputed days vs. observed).

A summary of the number of observed and imputed values has been added as follows:

L.185-187

*Per-fish, this resulted in 23.9 ± 9.9 and 26.0 ± 6.1 measured observations (mean ± standard deviation) from river entry to exit in 2017 and 2018, respectively, and 14.0 ± 7.8 and 8.4 ± 8.5 imputed values.*

Lines 183-185 (R1): As noted above, given the novelty of dynamic time warping in ecological studies (e.g. line 384) I think a paragraph or so explaining DTW (assumptions and basically what’s going on under the hood) is warranted.

Paragraph added as described above.

Line 189 (R2): I am not seeing blue and red time series in Figure 2, as described here in the figure caption, just shades of brown.

Figure 2 caption has been adjusted from blue/red to light/dark brown. This was a holdover from a previous iteration of the figure; thank you for bringing it to our attention.

Line 199 (R1): The language here is a little unclear to me because the authors do attempt to identify environmental drivers in 2018. I think some text is needed to clarify that the 2017 centroids were assumed to be correct. If this is the case though, it’s not clear to me how individuals that strayed (i.e. identified to one reach in 2017 and the other in 2018) were accounted for in this analysis. Additional clarification on this would be helpful.

The assumption is specified “…under the premise that the 2017 classifications best represented the two runs.” We have included more information on 2017 and 2018 samples used in this analysis.

L.226-227

*All classified individuals were included in 2017, and only those individuals correctly classified included for 2018.*

Line 213 (R2): Can leave out “excluding May”

Statement excluded.

Lines 248 (R1): I find the use of the term “individual spawning runs” a bit misleading because I most generally think of runs as describing behavior of multiple fish. Hence an individual run suggests, to me, a single categorical classification within a group (e.g. fall rather than spring or summer run), rather than an individual’s behavior. I would suggest replacing with individual migration phenologies, individual migrations, or a similar term as appropriate. Similarly, I think it may be more appropriate to phrase the following sentence as: “The median period of the first run, as defined by centroid 1, is…”

Thanks for these suggested changes they are now included. The “individual run” phrase has been eliminated throughout the manuscript.

Line 261 (R1): Any reason not to include river km, instead of latitude, as the y-axis? It seems to be more commonly referenced in the text than latitude (e.g. line 297), is a bit more intuitive to readers, and presumably it would be an easy swap given the data necessary to make Figure 1. Also spawning is mis-spelled.

We strongly agree with the reviewer that analyzing the spawning runs based on latitude is less-intuitive to readers than using river kilometer – this is even true for us and, as noted by the reviewer, reflected in our reference to “river kilometer” much more than “latitude”. Because of this, all analysis, figures, and text which had utilized or referenced latitude have been converted to river kilometer. As the Hudson is a linear north-south system, no results or inferences were affected by the change from latitude- to river-kilometer-based clustering.

Lines 268-269 (R1): To clarify, the median periods described here are defined by the median centroids identified for 2018; however these are not shown in in Figure 4 or anywhere else, correct? Personally, I think it might be better to show the 2018 centroids in the lower panels of Figure 4, since these seem to be of more interest than the misclassification patterns (which can be easily described in the text). If this change is made then I believe Table 1 could be removed.

The centroids (bold black lines) are classified for 2017 and applied to 2018 data. Note that these median phenologies are repeated across top and bottom rows. Individual phenologies are presented on top of these for both years. We strongly prefer to retain this presentation as it represents the full data set and allows the reader to see how the analysis operates. Similarly Table 1 is quite important in cross referencing narrative on run phenologies. The figure legend has been redrafted for clarity.

***Fig 4. Dynamic time warping centroid identification of Hudson River spawning runs.*** *Centroids are fitted to 2017 spawning run behaviors in both 2017 and 2018 (bold black lines; note centroid phenology is duplicated for both years). Individual phenologies are shown as gray and red lines, the latter indicating mis-classified individuals. Colored rectangles identify the river kilometers of the upper (red) and lower (blue) reaches.*

Line 293 (R2): I see a leveling off in Fig. 6 that starts the beginning of May and extends through mid-May, rather than a plateau only during mid-May

Text changed to “early May.”

Line 298 (R1): Again, it may be nitpicky but I would recommend defining run as a phenotype or suite of migration behaviors, rather than an observation. I.e. the authors studied two runs in two years, rather than four runs. As the authors note in their introduction, interannual stability is a defining characteristic of migratory contingents so it seems inappropriate to consider these as independent replicates.

Point taken, in response to earlier comments the reference to four runs was omitted. As indicated above we also omitted reference to individual runs.

Lines 315 (R1): The phrase 2017-categorized is a little confusing initially. In the case of 2016 individuals, I take it to mean fish that were tagged in the lower portion of the river and then had a centroid 2 type behavior?

As indicated in text, we could not assume tagging location was necessarily informative of run behavior in 2016. In this analysis 2016 data was classified according to the 2017 centroid analysis. We have added text to be more specific about this “reclassification.”

L.341-345

*In 2016, we reclassified individuals to spawning run contingent on the basis of the 2017 centroid analysis rather than tagging location and observed that the lower reach contingent departed the Hudson River estuary 6 days earlier than the upper reach contingent, similar to what was observed in 2017 when the lower reach contingent departed 9 days earlier on average.*

Line 359 (R1): The authors note that the midpoint of both runs in 2017 occurred at a similar temperature (1.7C difference). In 2018 it was a 1.8C difference. So I’m not sure the authors show strong evidence of a differential response to temperature given that interannual variability in temperature seems to overwhelm differences in the temperatures each run experienced.

A valid point. We have changed this statement to read – “and exhibited characteristic phenologies.” without reference to temperature cues.

Lines 360 (R1): Use of persist in this context seems a little out of place.

Word changed to participate.

Lines 361-363 (R1): The authors don’t present any data that allows for natural mortality to be distinguished from fishing mortality (e.g. tags returned by harvesters). I would recommend either removing the emphasis on harvest or that the authors add citations demonstrating that the majority of adult mortality is associated with harvest. If the latter, I would still suggest adding qualifying language given our generally poor understanding of natural mortality rates.

Accept the cautionary comment. In this introductory paragraph, we’ve opted for more generic text:

L.395-396

*These differential demographic fates between spawning runs can alter population outcomes, particularly if selective mortality occurs for one or the other contingent.* Then pick up greater explanation of likely fishing mortality effects later in the Discussion.

Lines 368-369 (R1): I’m not sure I follow. I assume the authors mean that first-spawners adopt the behaviors of older individuals that are proximate when migrations begin. However, the two runs do not appear to be segregated spatially or temporally during coastal residence and upstream migrants necessarily bypass downstream spawning habitats so it’s unclear to me where proximity and abundance come into play. I recommend the authors refine this hypothesis a bit more.

Line 370 (R1): Strictly speaking, this doesn’t seem to be an alternative hypothesis, but rather an explanation for how distinct behaviors are maintained (while the former seems to be more focused on how they arise).

In reference to the two preceding comments: Thanks for request for additional clarification and help specifying different parts of intended hypotheses. We have revised this section accordingly.

L.401-405

*For first maturing individuals, associative schooling behaviors with larger and older individuals within the Hudson River estuary could promote initial adoption of either spawning run behavior [29, 58, 59]. Thereafter spawning reach fidelity may relate to key environmental differences in conductivity, tidal flow, bathymetry and channel dimensions that allow piloting and then navigation to particular spawning reach features.*

Line 373 (R1): Insert “behavioral” (or synonym) before repertoire.

So revised.

Line 383-384 (R2): The authors included this statement: “Developed as a machine learning tool in speech recognition applications, we believe this is its [dynamic time warping] first application in comparing animal movement patterns.” As I wasn’t familiar with dynamic time warping, I did a quick search to see that it has recently been a suggested approach for analysis of animal movement as demonstrated within Cleasby et al. (2019), and applied to seabirds. I realize this is a recent paper, so understandably missed. I suggest the authors include this reference within the manuscript and adjust their statement accordingly.

Great find, which led us to a couple other papers on the topic. We were pleased to have support for this approach in the avian literature. We have included it in the following revised statement.

L.416-417

*Developed as a machine learning tool in speech recognition applications, it has received scant attention by movement ecologists[57]*

Line 390 (R1): What’s meant by comparable here?

Changed to overlapping.

Lines 429-430 (R1): Again nitpicky, but it seems that by definition spawning runs will exhibit differential use (temporal or spatial) of a habitat. Suggest rephrasing to something like: “Here we show that Hudson River striped bass exhibit evidence of distinct spawning runs despite broadly overlapping coastal distributions”.

Thanks for revision. Changes so made.

Line 436 (R1): I believe this should be Fig 3, however I also thought that Fig. 3 only provided an example comparison of two individuals. If so, then it seems like it would be better to reference Figure 4 and Table 1 (if retained). More generally I find the wording of this sentence confusing. It seems to imply that the lower run in 2017 is the only group to encounter the coastal fishery, which is clearly not the case. I would recommend rephrasing and moving the component on in-river vs. coastal slot limits to a separate sentence.

Greater distinction between 2017 and 2018 in terms of possible fishery impacts were made in response to earlier comments. We have made revisions regarding a separate statement on in river fishing. Thanks for catching the figure mis-reference.

Line 436 (R1): Outmigration data from 2016 are not clearly presented in the results, except in Figure 7 which is difficult to interpret at fine temporal scales. I’d recommend adding somewhere if they’re going to be discussed in detail.

Results on 2016 outmigration are made more specific in response to an earlier comment and are repeated here below.

L.341-345

*In 2016, we reclassified individuals to spawning run contingent on the basis of the 2017 centroid analysis rather than tagging location and observed that the lower reach contingent departed the Hudson River estuary 6 days earlier than the upper reach contingent, similar to what was observed in 2017 when the lower reach contingent departed 9 days earlier on average.*

Line 444-447 (R1): I’d finish this paragraph off by clearly stating that the similar coastal distributions suggest that differential survival in 2017 is likely driven by early ocean entry phenology

Change made to capture this. Thanks for improvement to paragraph.

Figure 7 (R1): I would recommend increasing the resolution on the final figure version and removing the panel borders that separate different years of observation. I think it would be better to show this as a one-panel figure with different years denoted along the x-axis so it’s immediately clear to readers that these are year-round observations (rather than 3 distinct tag deployments).

The figure has been altered as requested and combined with yearly maps of coastal receiver detections.

Table 1 (R1): I found Table 1 a little time consuming to interpret. I would recommend replacing with a figure providing equivalent summary statistics for easier interpretation. Alternatively, Figure 4 could be expanded to clearly show centroids in 2018, which would serve a similar purpose.

We respectfully request that the table is retained given that in contains specific dates that would be less precisely conveyed on a figure (which we’re assuming would look like a time line of some sort). We have changed the heading Median Entry to 50% Incidence to better align with emphasis on that statistic in text.
