# Supplementary material for: Multiple spawning run contingents and population consequences in migratory striped bass Morone saxatilis
Source: PLoS One. 2020 Nov 25;15(11):e0242797. doi: 10.1371/journal.pone.0242797 (PMC7688118; doi:10.1371/journal.pone.0242797)
Supplement: Attachment — Submitted filename: Response to Reviewer_Oct26.docx [file pone.0242797.s003.docx]

26 October 2020

Dear Editor:

All changes have been made according the reviewers helpful comments and edits.

Reviewer #1: Again, I greatly enjoyed the opportunity to read this manuscript and commend the authors on their work. They thoroughly addressed my original comments and I particularly appreciated the additional clarity regarding DTW. I only have a few additional suggestions listed below.

Line 33-35: Some awkward wording in this sentence.
Line 354: Contingents mis-spelled as continents.
Line 357-359: Check edits.
Figure 7. The figure has been updated to include receiver detections in the authors’ reply (bottom), but not in the attached figure preceding the revised manuscript. Editorial office should double-check before proofing.
Line 431: Suggest inserting a but before used to contrast the contingents differences from their similarities.
Line 432-438: Suggest rephrasing to clarify that in one of two years the contingents were exposed to early summer fisheries at different rates, which was associated with greater mortality.
Line 531: Add “with” before catch and release.
